# Combination Therapy of Metadichol Nanogel and Lipocalin-2 Engineered Mesenchymal Stem Cells Improve Wound Healing in Rat Model of Excision Injury

**DOI:** 10.34172/apb.2022.059

**Published:** 2021-09-12

**Authors:** Zahra Pourmohammadi-Bejarpasi, Reza Sabzevari, Amaneh Mohammadi Roushandeh, Ammar Ebrahimi, Mohammadreza Mobayen, Ali Jahanian-Najafabadi, Abbas Darjani, Mehryar Habibi Roudkenar

**Affiliations:** ^1^Medical Biotechnology Department, Paramedicine Faculty, Guilan University of Medical Sciences, Rasht, Iran.; ^2^Cellular and Molecular Research Center, Medicine Faculty, Guilan University of Medical Sciences, Rasht, Iran.; ^3^Burn and Regenerative Research Center, Medicine Faculty, Guilan University of Medical Sciences, Rasht, Iran.; ^4^Department of Pharmaceutical Biotechnology, School of Pharmacy and Pharmaceutical Sciences, Isfahan University of Medical Sciences, Isfahan, I.R. Iran.; ^5^Skin Research Center, Department of Dermatology, Razi Hospital, School of Medicine, Guilan University of Medical Science, Rasht, Iran.

**Keywords:** Mesenchymal stem cells, Metadichol, LCN2/NGAL, Wound healing, Excision injury

## Abstract

***Purpose:*** Currently, several disorders including burns, trauma, excisional and diabetic wounds, and bedsores threaten the human health. Application of mesenchymal stem cells (MSCs) is recommended for treatment of skin disorders. However, because of oxidative stress and inflammation after skin injury, survival of transplanted MSCs is low which in turn negatively affects the efficiency of the MSCs-based therapy. In an attempt to address the aforementioned challenge and introducing a novel potential therapeutic strategy, we employed combination therapy by lipocalin 2 (Lcn2)-engineered MSCs and a Metadichol (an inverse agonist of vitamin D receptor (VDR)) nanogel in a rat model of excisional wound.

***Methods:*** First, human umbilical cord MSCs (hUC-MSCs) was transfected by a recombinant plasmid encoding Lcn2 gene. Next, a combination of Metadichol nanogel and the engineered MSCs was co-applied on wound in rat model of excision injury. Finally the improvement of wound healing in experimental groups was evaluated by photography and histological assessments (hematoxylin and eosin staining).

***Results:*** Our findings revealed that the repair rate was higher in the group received combination therapy comparing to control groups. Notably, Metadichol+Lcn2-MSCs showed significantly higher wound contraction rate compared to control group at all time points (*P* value < 0.001). Furthermore, wound repair rate was 95% 14 days after surgery, and 100% after 21 days in the treatment groups. Our results also revealed that the combination therapy improved and accelerated the wound healing process.

***Conclusion:*** Our findings suggest a novel potential therapeutic strategy i.e. Lcn2-engineered MSCs and Metadichol for wound healing. However, further preclinical and clinical studies are required.

## Introduction

Skin is the largest organ of the body that protects it against mechanical and chemical insults. Furthermore, it provides innate and adaptive immune defenses and thermal regulation. It also plays important role in vitamin D synthesis and acts as a sensory organ.^1^ Skin injuries such as excisional wounds, diabetic wounds, bedsores, trauma, and burns are serious health problems that not only result in loss of all or parts of the skin and other tissues, but also could damage the defense barrier and lead to infection.^2-4^ Although some current therapeutic modalities for skin injuries (e.g. surgery and injectable dermal fillers) are useful to accelerate wound healing, they are often painful, and in most cases inefficient and unstable. Recently, some modalities such as mesenchymal stem cells (MSCs), gene therapy, and tissue engineering have been proposed for the treatment of cutaneous skin injuries.^5-10^ MSCs are capable of self-renewing, homing, tissue repair and regeneration, releasing trophic factors, promoting neovascularization, managing oxidative stress, and triggering anti-inflammatory responses.^11-14^ However, despite the mentioned advantages, MSCs-based therapy has some limitations including inadequate proliferation, limited post-transplantation viability, and gradual loss of their original characteristics during proliferation in a laboratory settings, as well as induction of apoptosis and cell death. In addition, the toxic and inflammatory microenvironment in recipient tissue is not in favor of the MSCs survival. It has been shown that over 99% of MSCs die within a few days post-transplantation.^15,16^ The major cause of this low survival rate is the inevitable exposure of the cells to a variety of stresses during preparation and following transplantation, including a nutrient-poor environment, oxidative stress, hypoxia, and high amount of cytotoxic factors.^17^ Hence, development of safe and efficient strategies in this regard has been under the focus of investigations and various strategies have been proposed to address the issue. Pre-treatment and/or preconditioning of MSCs with some growth factors and cytokines, H2O2, hypoxia, serum deprivation, and genetic engineering of MSCs by cytoprotective factors are examples of the strategies.^6^ Furthermore, arming MSCs with cytoprotective factors and/or natural substances harboring potential anti-oxidant and anti-inflammatory properties would be one of the strategies to address the challenges.^18,19^ Neutrophil gelatinase-associated lipocalin (NGAL) is a 25-kDa protein that belongs to the Lipocalin superfamily. Lipocalin 2 (Lcn2) is highly expressed in many cell types in response to a variety of stresses including oxidative stresses and inflammation, which are well-known stimuli in skin injury. Although the precise role of Lcn2 remains to be determined, as a well-known cytoprotective factor it has a variety of functions including anti-inflammatory, anti-oxidant, and anti-bacterial properties.^20-23^

Recently, a new natural product (NP) named Metadichol was designated a “generally recognized as safe (GRAS)” substance by the United States Food and Drug Administration (FDA).^24^ Metadichol is a nano-formulation of long-chain lipid alcohols (policosanol) derived from a variety of sources including wheat, rice, distillers grains, sugar cane, grape, sorghum, apples, and peanuts.^25-29^ It is an inverse agonist of vitamin D receptor (VDR), aryl hydrocarbon receptor, and ROR gamma nuclear receptor which their beneficial effects in skin disorders have been shown previously.^24^ Interestingly, it has been also reported that Metadichol has anti-oxidant and anti-inflammatory properties.^30^

Considering the limitation of MSCs-based therapy and also the positive effects of Metadichol on wound healing, this study was conducted to explore the therapeutic potential of a novel modality including combined topical application of Metadichol and administration of Lcn2 engineered MSCs in a rat model of excision injury. Here we used a commercially available Metadichol® nanogel to mimic a wound dressing hydrogel which provides moisture and absorbs exudate. As we report here, the combination therapy exhibited improved wound healing capability compared to controls. Our findings suggest that the combination therapy might be considered as a novel therapeutic strategy for wound healing.

## Materials and Methods

### MSCs isolation and culture

Human umbilical cord MSCs (hUC-MSCs) were isolated as previously described.^5^ The cells were cultured in DMEM low glucose and 10% FBS at 37°C and 5% CO_2_ (All the materials purchased from Gibco, Germany).

### Overexpression of Lcn2 in hUC-MSCs

hUC-MSCs were transfected with 3 μg/mL of either the pcDNA3.1/CT-GFP-Lcn2 (Lcn2-MSCs) or the pcDNA3.1/CT-GFP empty vector (V-MSCs) using X-tremeGENE HP DNA transfection kit (Roche, Germany) according to the manufacturers’ protocol and as described previously.^31^

### RNA extraction and RT-PCR analysis of the Lcn2 mRNA expression

Total RNA of the transfected cells was extracted by TRIzol reagent (Invitrogen, USA) according to the manufacturer’s instructions and quantified using NanoDrop ND-1000 (NanoDrop, USA) spectrophotometer. Next, 2 μg of the extracted RNA was used for cDNA synthesis using SuperScript VILO cDNA Synthesis Kit (Invitrogen, USA) according to the manufacturer’s instructions. Polymerase chain reaction (PCR) was performed by a T100^TM^ Thermal Cycler (Bio-Rad, USA). PCR condition included a primary denaturation step at 95°C for 5 minutes, followed by 35 cycles of PCR (95°C for 30 seconds, 57°C for 30 seconds, and 72°C 30 seconds), and a final step at 72°C for 5 minutes. Lcn2 primer pair included forward: 5′-TCACCTCCGTCCTGTTTAGG-3′ and reverse 5′-CGAAGTCAGCTCCTTGGTTC3′. Β-actin was considered as an internal control and amplified using a primer pair including forward: 5′-TTCTACAATGAGCTGCGTGTGC-3′ and reverse 5′-GTGTTGAAGGTCTCAAACATGAT-3′. All primers were designed by Primer 3 software and synthesized by Bioneer Company (South Korea). PCR products were electrophoresed on 2% agarose gel.

### Assessment of the Lcn2 protein expression

Expression of LCN2 at protein level was measured by an ELISA kit (R&D Systems, USA). Concisely, 48 hours post-transfection, the cell culture medium of the Lcn2-MSCs and V-MSCs was collected, centrifuged at 1500 rpm for 5 minutes to remove cells and debris, and used for measurement of the Lcn2 content.

### In vivo tracking of hUC-MSCs by CellTracker^TM^ CM-Dil

The hUC-MSCs were detached by trypsin-EDTA solution and pelleted by centrifugation at 1500 rpm. Then, 1×106 of the cells were resuspended in 1µg/mL of CellTracker^TM^ CM-Dil dye (Thermo Fisher Scientific, US) and incubated at 37°C, 5% CO2 and 90% humidity for 5 minutes, followed by incubation at 4°C for 15 minutes. The cells were washed twice with phosphate-buffered saline (PBS) and finally resuspended in 150 µL PBS for direct injection into the periphery of the excision wound. The animals were sacrificed 4 and 7 days after the injection and the skin was removed for tissue processing. Briefly, following fixation in 10% formaldehyde, the tissues were paraffin-embedded and 3 µm sections were prepared by manual microtome. The sections were deparaffinized and stained by 1 µg/mL DAPI (Sigma, USA) for nuclear staining. Finally, the transplanted cells were tracked under a fluorescent microscope (Nikon, Japan).

### Animal study

Seventy-two male Wistar rats weighing 200-220 g were used in the present study. All animal handlings and protocols were approved by the Guilan University of Medical Sciences ethical committee for animal studies. The animals were kept in a standard animal house with ambient temperature around 20-22°C, 12 hours/12 hours dark/light cycles, and free access to food and water.

### Excisional wound model

The rats were anesthetized by intraperitoneal injection of Ketamine (Alfasan, Netherlands) (70 mg/kg) and xylazine (Alfasan, Netherlands) (7 mg/kg). The dorsal skin of the animals was shaved and disinfected with 70% ethanol. Next, a full-thickness excisional wound (1.5 cm diameter) was made on the skin using a sterile surgical punch. Then, 150 µL of the cell suspension was subcutaneously injected at four points which were 3 mm away from the wound edge. Simultaneously, 0.4 g Metadichol nanogel (Generation100 Company, Switzerland) was applied to the wound and covered by a transparent wound dressing (Comfeel®, Coloplast, Denmark) to prevent infections. A circular ring was sutured around the wound to inhibit panniculus carnosus muscle contraction. The animals were divided to 6 groups (n = 3) including control (Ctrl; the rats with excision wound without any treatment), Metadichol (Met; the rats with excision wound that received Metadichol) V-MSCs (V-MSC; the rats with excision wound that received the V-MSCs ), Lcn2-MSCs ( Lcn2-MSC; the rats with excision wound that received the Lcn2-MSCs), V-MSCs and Metadichol (Met+V-MSCs; The rats which received both topical Metadichol and V-MSCs injection), Lcn2-MSCs and Metadichol (Met+Lcn2-MSCs; The rats which received both topical Metadichol and Lcn2-MSCs injection).

### Measurement of wound healing

Wound area was photographed at 0, 4, 7, 14, and 21 days after surgery, and their healing rate was measured by ImageJ software (NIH, USA, version 1.8.0-112). The percentage of wound closure was calculated using the following formula:


Wound Contraction00=Initial Wound Size−Specific Day Wound Size/Initial Wound Size×100

### Histopathological studies

The rats were sacrificed by an overdose of ketamine/xylazine. The wound area and its adjacent normal skin were removed 4, 7, 14, and 21 days after surgery and fixed in 10% formaldehyde at least for 72h, and then dehydrated through a series of graded ethanol (70%, 90%, 96% and 100%). Then, the tissues were paraffin-embedded and 5 µm sections were obtained by manual microtome. Next, the slides were stained with hematoxylin and eosin dyes according to routine laboratory protocols. In addition, the formation and arrangement of collagen fibers were evaluated by Masson’s trichrome staining of different groups 14, and 21 days after surgery.

### Statistical analysis

All data were analyzed by GraphPad Prism software version 8.0.2 (NIH, USA). Wound closure was analyzed using the linear regression method. In addition, the *t* test, two-way ANOVA, and Tukey’s post hoc for multiple comparisons test were used. The Shapiro-Wilk test was used to examine the normal distribution of the data. Furthermore, One-way ANOVA and Kruskal-Wallis statistical tests were used to analyze the results, and *P* < 0.05 were considered significant.

## Results

### Overexpression of Lcn2 in hUC-MSCs

**Figure 1:**
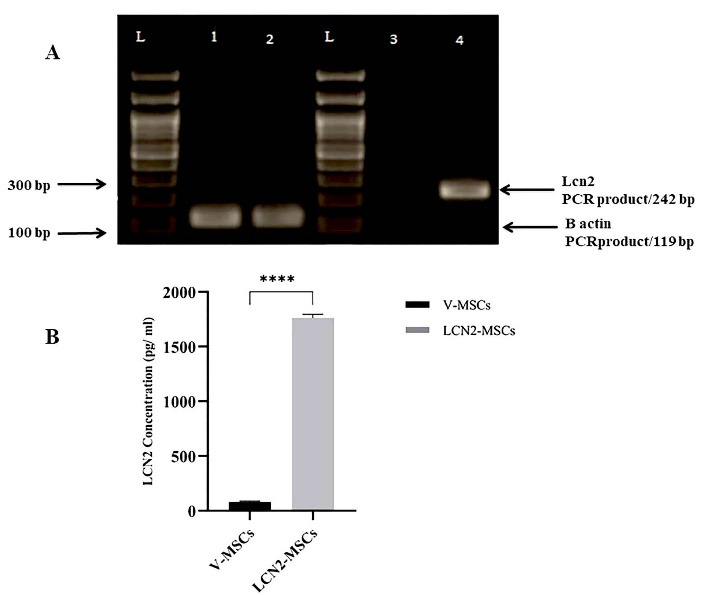
Evaluation of Lcn2 overexpression at mRNA and protein levels. (A) RT-PCR was performed to evaluate the expression of Lcn2 mRNA. Detection of a single band of 242 bp in the Lcn2-MSCs group and weak PCR product in V-MSCs indicated successful expression of Lcn2. Expression of β-actin was used as an internal control. Lane 1 = β-actin of V-MSCs, lane 2 = β-actin of Lcn2-MSCs, lane 3 = Lcn2 of V-MSCs, lane 4 = Lcn2 of Lcn2-MSCs). (B) ELISA was performed to assess Lcn2 at the protein level. The culture medium of V-MSCs and Lcn2-MSCs were harvested 48 h after transfection. The Lcn2 protein expression was significantly (*P* < 0.0001) higher in Lcn2-MSCs compared to the V-MSCs. **** *P* < 0.0001, (Number of replicates = 4).

Overexpression of Lcn2 at mRNA and protein levels were verified by RT-PCR and ELISA, respectively. A sharp band of 242 bp on the 2% agarose gel was detected in the Lcn2-MSCs, while a faint band was noticed in the V-MSCs (Figure 1A). Overexpression of Lcn2 protein by the Lcn2-MSCs was confirmed while the expression amount was low in the V-MSCs) (Figure 1B).

### Metadichol increased the hUC-MSCs survival following their transplantation to the wound

**Figure 2:**
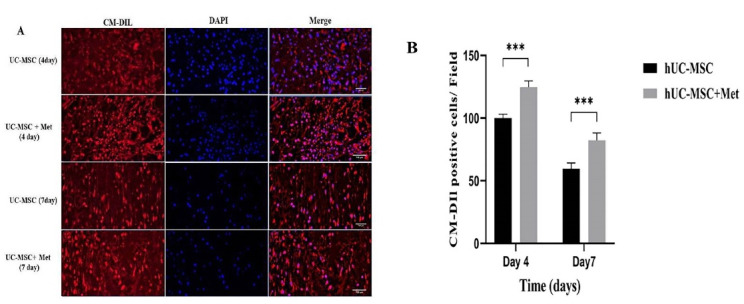
*In vivo* tracking of the hUC-MSCs in excision wound 4 and 7 days after transplantation. The cells were labeled by CellTracker^TM^ CM-Dil before transplantation. (A), Higher numbers of red-stained cells were identified in the hUC-MSCs+Met group comparing to the hUC-MSCs group. In addition, the cells’ nuclei were stained by DAPI and the photographs were merged. the hUC-MSCs group received the labeled cells, the hUC-MSCs+Met group received the labeled cells and Metadichol as well (B), The percentage of the CellTracker^TM^ CM-Dil labeled hUC-MSCs in wound area on 4th and 7th days post-transplantation. The hUC-MSCs group received the labeled cells, the hUC-MSCs+Met group received the labeled cells, and Metadichol as well; ***P ≤ 0.0001. Number of replicates = 3.

To track the hUC-MSCs following transplantation, they were first labeled by CellTracker^TM^ CM-Dil and then administered to the animals. As it is represented by Figure 2A, the presence of the labeled hUC-MSCs in the wound area was confirmed on days 4 and 7 post-transplantation in the hUC-MSCs and hUC-MSCs+Met groups. In addition, the number of the cells in the hUC-MSCs+Met group was significantly (*P* < 0.001) higher than the hUC-MSCs on day 4. Furthermore, although the number of the cells in both groups gradually decreased within one week, it was still significantly (*P* < 0.001) higher in the hUC-MSCs+Met group (Figure 2B).

### Combination therapy of Metadichol and Lcn2-MSCs effectively improved wound healing macroscopically

**Figure 3:**
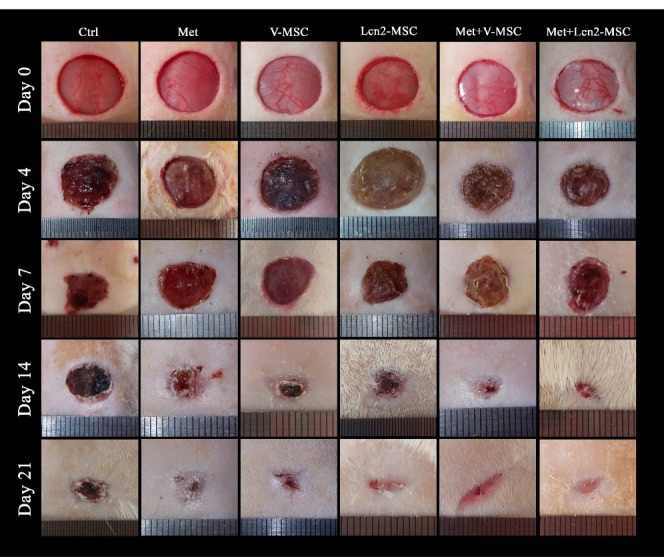
Photographs of wound contraction at different time points after excisional wound model in different groups. Control (Ctrl; the rats with excision wound without any treatment), Metadichol (Met; the rats with excision wound that received Metadichol), V-MSCs (V-MSC; the rats with excision wound that received the V-MSCs ), Lcn2-MSCs ( Lcn2-MSC; the rats with excision wound that received the Lcn2-MSCs), V-MSCs and Metadichol (Met+V-MSC; The rats which received both topical Metadichol and V-MSCs injection), Lcn2-MSCs and Metadichol (Met+Lcn2-MSC; The rats which received both topical Metadichol and Lcn2-MSCs injection).

**Figure 4:**
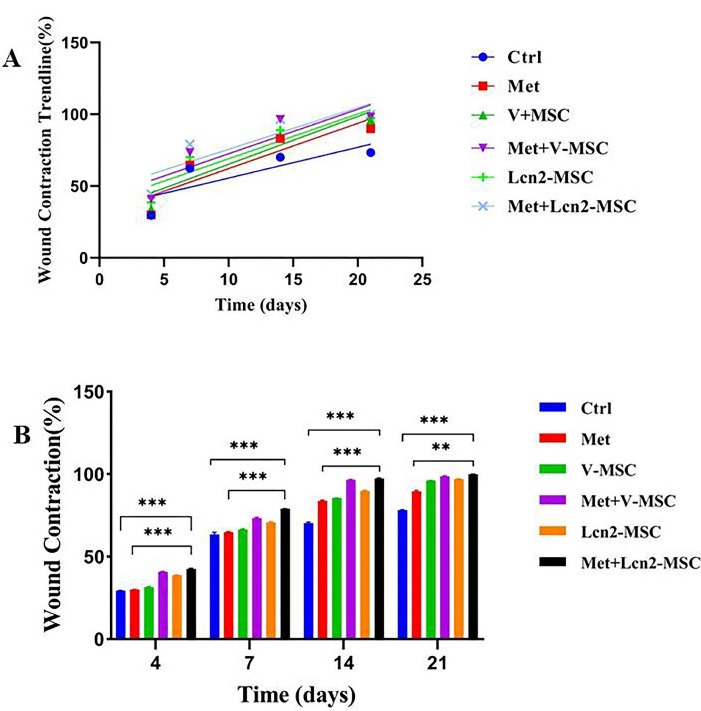
Wound healing rate in different groups, illustrated as linear regression and wound repair comparison among groups. (A) Linear regression analysis of wound healing rates. The slope of each line expresses the wound healing extent. (B) Wound contraction rate in different groups using ANOVA test. Control (Ctrl; the rats with excision wound without any treatment), Metadichol (Met; the rats with excision wound that received Metadichol), V-MSCs (V-MSC; the rats with excision wound that received the V-MSCs ), Lcn2-MSCs ( Lcn2-MSC; the rats with excision wound that received the Lcn2-MSCs), V-MSCs and Metadichol (Met+V-MSC; The rats which received both topical Metadichol and V-MSCs injection), Lcn2-MSCs and Metadichol (Met+Lcn2-MSC; The rats which received both topical Metadichol and Lcn2-MSCs injection) *** *P* < 0.001, ** *P* < 0. 01. Number of replications = at least 3.

The wound contraction area was photographed to evaluate its healing and closure on days 0, 4, 7, 14, and 21. The process of wound healing in macroscopic view and its repair rate is represented by Figures 3 and 4, respectively. The first progress in wound healing was observed on day 7, in which the Lcn2-MSC, Met+V-MSC, and Met+Lcn2-MSC groups showed more contraction compared to the other groups. In addition, a great progress in wound healing and contraction occurred from days 7 to 14 in the treatment groups. On day 14, 90% of the wound area was healed and closed in the Met+V-MSC and Met+Lcn2-MSC groups. It is noteworthy that on day 21, wound contraction was more than 90% in all groups except control, however, the Met, Lcn2-MSC, Met+V-MSC, and Met+Lcn2-MSC groups showed nearly 100% wound closure (Figure 4A). In addition, the control group showed the lowest contraction rate among all groups at this time point. Overall, wound healing was prominent in the Met+Lcn2-MSC group for 21 days (Figure 4B). Moreover, the wound area contraction rate was measured by Image J software and the results are represented by Figure 4 (A-B). According to Figure 4A, the wound closure rates were not significantly different among the experimental groups. However, all groups showed a higher rate of wound closure when compared to the control group (*P* < 0.001). Of note, the wound area size in the Met+Lcn2-MSC group was significantly smaller than the other groups from day 7 to 21 (*P* < 0.001). It is worth mentioning that the wound healing rate was significantly higher in the Met+ Lcn2-MSC group (*P* < 0.001).

### Combination therapy of Metadichol and Lcn2-MSCs improved wound healing histologically

**Figure 5:**
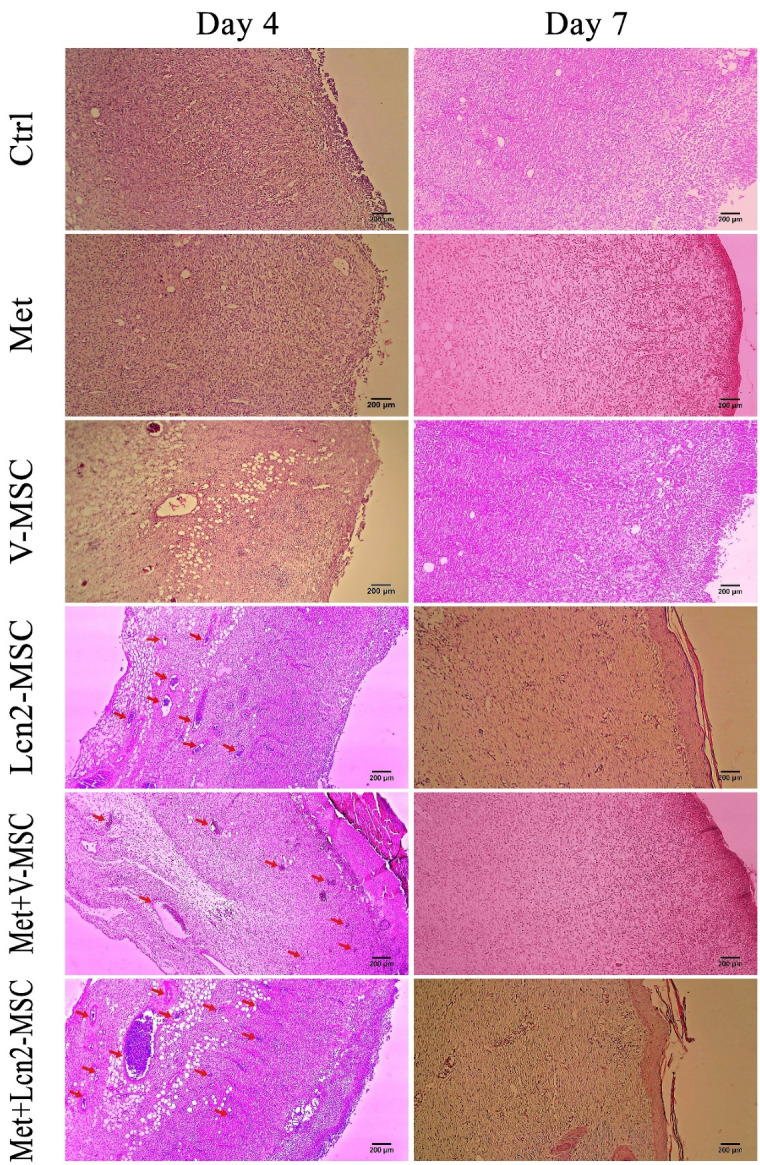
Photomicrographs of wound area histomorphology stained by hematoxylin-eosin 4 and 7 days after wound creation. Neovascularization (red arrows) in Met+Lcn2-MSC was higher than that in other groups on days 4 and 7. Of note, partial epithelization was detected 7 days after surgery in Met+Lcn2-MSC, Met+V-MSC, and Lcn2-MSC groups (yellow dots). Images are represented with 10x magnification. Control (Ctrl; the rats with excision wound without any treatment), Metadichol (Met; the rats with excision wound that received Metadichol), V-MSCs (V-MSC; the rats with excision wound that received the V-MSCs ), Lcn2-MSCs ( Lcn2-MSC; the rats with excision wound that received the Lcn2-MSCs), V-MSCs and Metadichol (Met+V-MSC; The rats which received both topical Metadichol and V-MSCs injection), Lcn2-MSCs and Metadichol (Met+Lcn2-MSC; The rats which received both topical Metadichol and Lcn2-MSCs injection) Scale bar = 200 μm.

**Figure 6:**
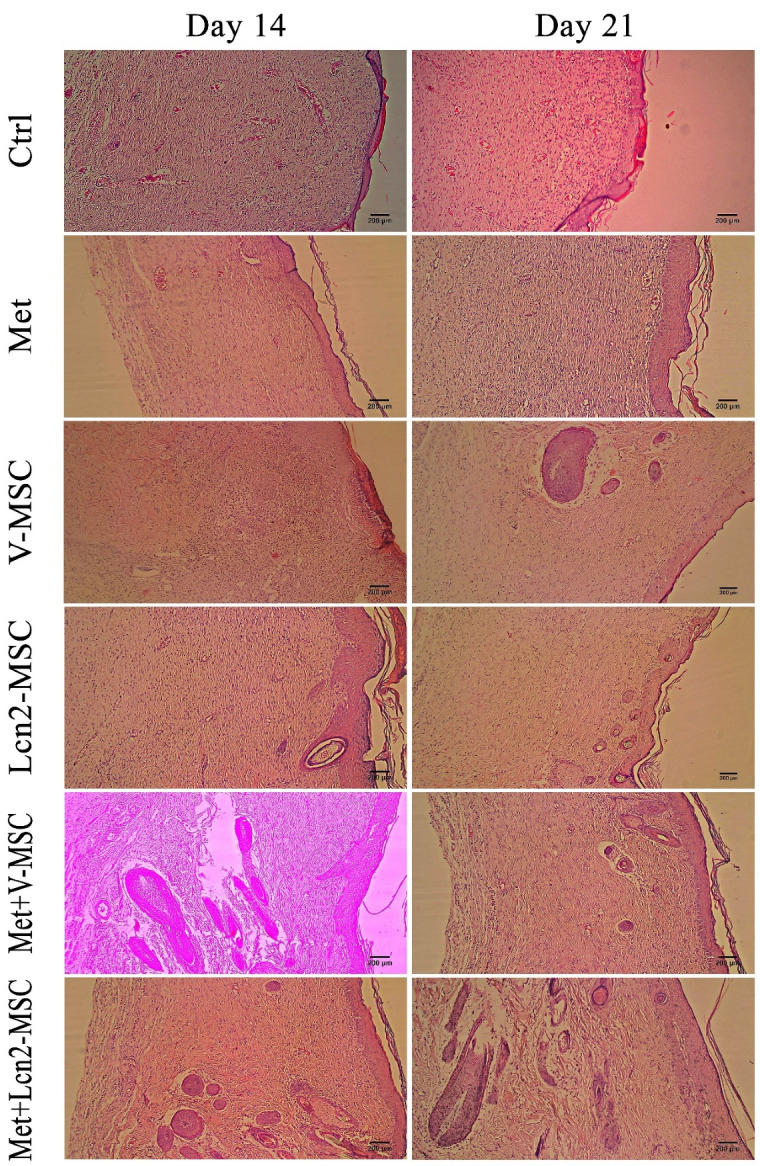
Photomicrographs of wound area histomorphology stained by hematoxylin-eosin 14 and 21 days after wound creation. Skin appendages like sebaceous glands were only detected In Met+ Lcn2-MSC and Met+V-MSC groups on days 14 and 21. Images are represented with 10x magnification. Control (Ctrl; the rats with excision wound without any treatment), Metadichol (Met; the rats with excision wound that received Metadichol), V-MSCs (V-MSC; the rats with excision wound that received the V-MSCs ), Lcn2-MSCs ( Lcn2-MSC; the rats with excision wound that received the Lcn2-MSCs), V-MSCs and Metadichol (Met+V-MSC; The rats which received both topical Metadichol and V-MSCs injection), Lcn2-MSCs and Metadichol (Met+Lcn2-MSC; The rats which received both topical Metadichol and Lcn2-MSCs injection). Scale bar = 200 μm.

**Figure 7:**
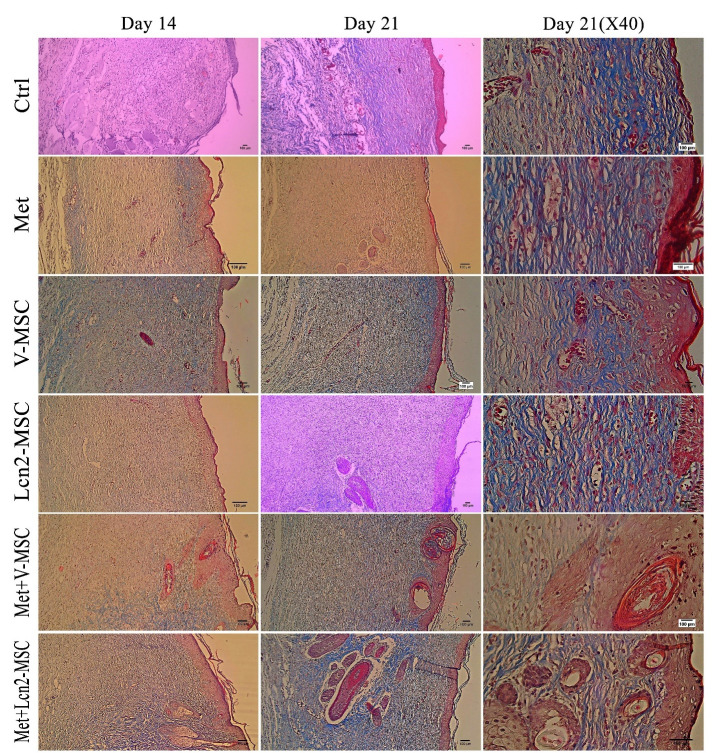
Photomicrographs of collagen arrangement assessed by Masson's trichrome staining 14 and 21 days after excisional wound creation. The collagen tissue was denser and more mature in all groups except the control group. In addition, collagen fibers have been distributed more regularly and densely throughout the wound area in the Met+Lcn2-MSC and Met+V-MSC groups on day 21. Collagen arrangement in Met+Lcn2-MSC and Met+V-MSC groups represented the highest quality and was well-designed compared to other groups. In the control group, non-uniform and irregular collagen thickness was observed. Control (Ctrl; the rats with excision wound without any treatment), Metadichol (Met; the rats with excision wound that received Metadichol), V-MSCs (V-MSC; the rats with excision wound that received the V-MSCs ), Lcn2-MSCs ( Lcn2-MSC; the rats with excision wound that received the Lcn2-MSCs), V-MSCs and Metadichol (Met+V-MSC; The rats which received both topical Metadichol and V-MSCs injection), Lcn2-MSCs and Metadichol (Met+Lcn2-MSC; The rats which received both topical Metadichol and Lcn2-MSCs injection) Scale bar = 100 μm.

Furthermore, the wound and its normal adjacent tissue were stained by hematoxylin and eosin at 4, 7, 14, and 21 days after surgery (Figures 5 and 6). In addition, the collagen architecture was evaluated by Masson’s trichrome staining (Figure 7). As it is shown by Figure 5, a large number of inflammatory cells were seen 4 days after surgery in the wound area, however, no significant difference was observed among the groups. In addition, hemorrhagic lesions were observed more in the control group. Furthermore, angiogenesis and a large number of vessels were observed in the Met+V-MSC, Met+Lcn2-MSC, and Lcn2-MSC groups. Of note, partial epithelization was detected 7 days after surgery in Lcn2-MSC, Met+V-MSC, and Met+V-MSC groups (Figure 5).

Our finding revealed that the configuration of the wound area was histologically different between the two-time points of 4 and 7 days post-surgery. It is noteworthy that granulation tissue with a dense and compact cell population clearly formed in all groups. However, the nucleation density was higher in the Met+Lcn2-MSC and Met+V-MSC groups. Furthermore, although the formation of the epithelium was thin, incomplete, and discontinuous in all groups, however, it was slightly well-differentiated and organized in Met, Met+V-MSC, and Met+ Lcn2-MSC groups. Moreover, a high rate of angiogenesis was still present in all groups, especially in the Met +Lcn2-MSC group. It is worth mentioning that granulation tissue and cell density were still observable in the dermis, though with a slight decrease, in most groups on day 14 (Figure 6). However, the Met+Lcn2-MSC group represented minimal cell density and normal histological configuration resembling healthy tissue. In addition, the quality of the epithelium was higher in all experimental groups compared to the control. In the control group, the epithelium was thicker and not well organized with vacuolated and larger keratinocytes. In addition, the skin appendage including sweat glands and hair follicles were seen in Met+Lcn2-MSC and Met+V-MSC groups. On day 21, a decrease in granulation tissue, cell population, and density were the prominent features in all groups. Interestingly, immature sweat glands and hair follicles were formed in Met+Lcn2-MSC, Met+V-MSC, V-MSC, and Lcn2-MSC groups as well (Figure 6).

The collagen architecture in wound tissue on day 14 is represented by Figure 7. As it is shown, the Met+Lcn2-MSC, Met and Met+V-MSC groups had denser, thicker, and more regular collagen fibers than the control group. In addition, the Met+Lcn2-MSC group showed the highest collagen quality in terms of thickness, organization, and density compared to all other groups. Furthermore, 21 days after surgery, the formation, and organization of collagen fibers were more compact at the edges of the wound, and from the margin of the wound to its center. Collagen was distributed uniformly throughout the wound in a thick and dense form and almost parallel to the wound area, with a regular distribution, especially in the Met+Lcn2-MSC group. In addition, the quality of collagen arrangement was higher in the Met+V-MSC and Met groups compared to the control.

## Discussion

Skin injuries including excisional wounds are amongst public health problems with a high annual death toll. ^32^ In this study, a multidisciplinary approach was employed for the treatment of wound healing in the rat model. We found that combination therapy by Lcn2-MSCs transplantation and Metadichol nanogel application accelerates wound healing in a wound excision rat model. Although the UC-MSCs transplantation has been shown to promote wound healing in previous studies, there have been few reports on its healing effect on excisional wounds.^33,34^ In the current study, we found that the number of transplanted UC-MSCs in wound tissues decreased gradually over time. This might be due to the harsh microenvironment of the excision injury. Supporting this notion, the toxic microenvironment of the recipient host, mainly due to inflammation and oxidative stress, showed to make an unfavorable microenvironment for MSCs to survive, and thereby, resulted in decreased viable cells and limited the capacity of the cell to exert their therapeutic effects within first days of the transplantation.^35,36^ Within our assumption, when we administered Metadichol nanogel and hUC-MSCs together, our results revealed that the number of the viable cells that were positive for CM-DIL were higher in the hUC-MSCs+Met group compared to the hUC-MSCs group suggesting that Metadichol might reduce the toxicity of the environment by its anti-inflammatory and anti-oxidative functions which enhances the efficiency of MSCs-based therapy.^25,26,28,35,36^ This might arise the fact that Metadichol, as an inverse agonist of vitamin D3, could mitigate the harsh microenvironment by its anti-oxidant and anti-inflammatory properties, therefore, the transplanted MSCs would survive longer.^24^ It has been reported that vitamin D3 is able to suppress Th-1 cell proliferation that in turn decreases the production of gamma interferon and interleukin-2. Furthermore, it has been well-known that vitamin D3 suppresses the production of IL-17 via the direct reduction in IL-17 gene transcription.^24-26,29,30^ Vitamin D3 also upregulates the expression of Th-2-associated cytokines such as interleukin-4 and interleukin-10. Overall, vitamin D3 regulates and switches the Th-1 responses toward Th-2 ones. However, the precise mechanisms underlying Metadichol effect on alleviating the unfavorable microenvironment for MSCs to survive after transplantation remains to be studied in the future.

In this study, we also found that the combination therapy resulted in an approximately normal histological configuration in skin resembling healthy tissue.^37,38^ Angiogenesis is an important factor especially in the early stages of wound healing which helps in proper nutritional support and removal of toxic substances.^39^ The rate of vessel formation is high in the early stages of wound healing, but is reduced at the final stages. Interestingly, it was clearly evident that the number of vessels was low in the Lcn2-MSC+Met group 21 days after wound creation. In addition, the re-epithelialization in the Lcn2-MSC+Met group was well organized. Lack of formation of skin appendices such as sweat glands and hair follicles is a major problem in the wound healing process. However, in our study, sweat glands and hair follicles were observed following combination therapy, particularly in the Lcn2-MSC+Met group.

The results of a recent study conducted by Dong et al reported that injectable and tunable gelatin hydrogels increased murine adipose‐derived stem cells (AD-MSCs) retention and enhance cutaneous wound healing in the rat.^40^

Based on their findings, complete wound closure lasted 28 days, whereas, in our study, it took 14 days for a complete wound closure to accomplish. In addition, Xu et al designed an injectable hydrogel system of hyperbranched multi-acrylated poly (ethylene glycol) macromers (HP-PEGs) and thiolated hyaluronic acid, as cell retention and delivery platform. Next, they tested the efficacy of the adipose-derived mesenchymal stem cells (AD-MSCs) embedded hydrogels for the treatment of diabetic wounds in a murine animal model. They revealed that angiogenesis was increased and the re-epithelialization was enhanced that supports our findings. Moreover, they reported the inhibition of inflammation after combination therapy in diabetic wound injury.^41^

Overexpressing cytoprotective factors and/or genes implicated in regeneration potential of skin in MSCs such as c-JUN, VEGF, PHD2, Wnt7a, LL37, and TGFβ3 have been reported previously.^42-44^

More recently, Yue et al overexpressed or silenced c-Jun in hUC-MSCs and investigated the therapeutic effects of engineered hUC-MSCs on wound healing in a diabetic rat model. They revealed that in situ subcutaneous injection of c-Jun-overexpressing hUC-MSCs increased angiogenesis and re-epithelialization, accelerated wound healing, and upregulated hepatocyte growth factor and platelet derived growth factor A protein expression in wound tissues. Despite the promising regenerative capability of the c-Jun-overexpressing hUC-MSCs, genetic manipulation of cells using viral vectors is challenging.^45^

In the current study, we overexpressed Lcn2 in UC-MSCs and with Metadichol nanogel co-applied on a wound to improve the wound healing rate. Harder and Schröder reported that NGAL/Lcn2 expression is low in healthy skin but is increased in diseases such as psoriasis due to inflammatory conditions.^46^

Lcn2 may facilitate macrophage polarization toward the M2 phenotype and improve impaired angiogenesis in wound inflammatory conditions. However, the precise role of Lcn2 in skin regeneration has not been fully understood yet and warrants further studies.^47,48^

One of the main critical processes in wound healing is re-epithelization. It is suggested that Lcn2 has a fundamental role in the migration of the epithelial cells in wound repair. Qi Miao reported that Lcn2 acts as the key secreted factor downstream of Tcf3. Furthermore, their findings revealed that Lcn2 enhanced cells migration toward wound and accelerated wound repair.^23^

In addition, it has been suggested that Lcn2 could improve cells migration via binding to and activation of the matrix metalloproteinase-9 (MMP-9).

In another study conducted by Wlian et al, the regenerative potential of a lipocalin-derived molecule was evaluated in wound healing. They revealed that this molecule plays role in wound healing via fibroblast modulation and extracellular matrix remodeling. It was suggested that this peptide increases the activity of matrix metalloproteinase 2 (MMP2) which results in cell migration and re-epithelization. It has been proven that MMP2 has a fundamental role in wound healing through tissue remodeling, extracellular matrix quality, migration, and adhesion molecules. Hopefully, lipocalin-derived peptide might be considered as a pharmaceutical agent in wound healing.^49^ Metadichol has also been used to treat other types of diseases, such as chronic kidney disease, and cancer, and heart diseases.^25-27,29,49^ Raghavan et al reported that administration of Metadichol in patients suffering from chronic kidney diseases with high levels of red cell distribution width resulted in improvement of a variety of biomarkers such as red cell distribution width, indicating the effectiveness of Metadichol.^30^

## Conclusion

In an attempt to improve the MSCs-based therapy for wound healing, we employed a novel modality by combining topical application of Metadichol and administration of Lcn2 engineered MSCs. Our results revealed that Metadichol nanogel increases the MSCs survival after transplantation, which could be due to anti-oxidative and anti-inflammatory properties of Metadichol. Lcn2 might also render anti-oxidant, anti-bacterial, anti-apoptotic, and anti-inflammatory properties which makes the MSCs to be refractory to the unfavorable microenvironment of the injured skin. Our findings also showed that the combination therapy improved and accelerated the wound healing process and resulted in well-organized epithelization, enhanced formation of skin appendices such as hair follicles and sweat glands, and improved arrangement of collagen. Although further preclinical and clinical trials are required in this regard, our findings suggest that the combination therapy might be considered as a novel potential therapeutic strategy for wound healing.

## Acknowledgments

This study was supported by the research deputy of Guilan University of Medical Sciences (Grant number; IR.GUMS.REC.1397.299).

## Ethical Issues

All experimental procedures were approved by the Institutional Animal Care and Use Committee (IACUC) at Guilan University of Medical Sciences.

## Conflict of Interest

The authors declare that they have no conflict of interest.
